# Assessment of Chokeberry Powders Quality Obtained Using an Innovative Fluidized-Bed Jet Milling and Drying Method with Pre-Drying Compared with Convection Drying

**DOI:** 10.3390/foods10020292

**Published:** 2021-02-01

**Authors:** Anna Sadowska, Franciszek Świderski, Ewelina Hallmann, Katarzyna Świąder

**Affiliations:** Department of Functional and Organic Food, Institute of Human Nutrition Sciences, Warsaw University of Life Sciences, Nowoursynowska Str. 159c, 02-776 Warsaw, Poland; franciszek_swiderski@sggw.edu.pl (F.Ś.); ewelina_hallmann@sggw.edu.pl (E.H.); katarzyna_swiader@sggw.edu.pl (K.Ś.)

**Keywords:** chokeberries, drying methods, powders

## Abstract

There is a need and great interest among food producers in obtaining powders from fruit and vegetables of both high nutritional value and sensory qualities superior to those hitherto obtained by convection drying (CD) and spray drying methods and cheaper to prepare than the sublimation method. This study is focused on whether powders can be obtained from fruit berries with a sticky structure, using the chokeberry (*Aronia melanocarpa*) as a test example, by a combined fluidized-bed jet milling and drying (FBJD) of pre-dried fruit by CD to an adequate water activity (a_w_). The pre-drying step reduced sticking between fruit particles during the simultaneous drying and grinding processes of the FBJD method in order to obtain powders of desired granulation. Three different pre-drying temperatures of 50, 60, and 70 °C were tested for levels of microorganisms in chokeberries at a water activity of 0.4. Vitamin C content and antioxidant properties were also examined along with polyphenol separation. Fruit pre-dried at 60–70 °C had significantly higher vitamin C and polyphenolic content and greater antioxidant properties than those pre-dried at 50 °C. Further studies were thus undertaken on powders pre-dried at 70 °C in which antioxidant properties, vitamin C, and polyphenols content were also compared with CD obtained powders. The FBJD method combined with CD pre-drying proved superior to just using the CD method, where powders had a greater preservation of vitamin C at 84% (CD powders 35%), a 12% higher total polyphenol content, and a 10% higher antioxidant activity. The test method also uses a much shorter drying time than the CD method, because the grinding of the hard-textured material takes only few minutes.

## 1. Introduction

There is increasing interest in using fruit and vegetable powders as sources of bioactive compounds, natural flavoring, or coloring additives to replace the widely used flavorings and dyes marked with the “E” symbol. It is even more desirable to prepare powders from the ‘superfruits’ grouping in the production of functional foods and dietary supplements in that they constitute sources of vitamin C, polyphenols, and carotenoids [1]. Fruit berries provide rich sources of many bioactive compounds with high antioxidant potential; these include the chokeberry, blackcurrant, strawberry, and raspberry. When used in a powder form for example, even a small amount ranging 30 to 70 g [1,2] can deliver the recommended daily dietary intake of vitamin C [3].

The black chokeberry (*Aronia melanocarpa*) is a native North American shrub belonging to the Rosaceae family. Its fruit are rich in polyphenols (819–2996 mg gallic acid equivalents/100 g fresh mass), with a strong antioxidant capacity that include anthocyanins, flavonols, flavanols, proanthocyanidins, and phenolic acids [4]. Anthocyanins are the main group of polyphenols in this fruit, and are responsible for the tart taste and intense color [5]. Many studies have shown these compounds to beneficially affect health [6]. Anthocyanins have been proved to benefit the circulatory system and heart function, stimulate insulin secretion, and improve the functioning of the retina for example [5]. Because of the chokeberry’s tart taste, it is however not usually eaten raw. This fruit therefore requires suitable processing to ameliorate its tart taste to make it more suitable for general consumption as a semi-finished or finished foodstuff product. A common process used for chokeberries is drying and then grinding to a powder consistency, thereby allowing incorporation into a wide range of products, such as teas, drinks, yoghurts, desserts, or dietary supplements [7].

The bioactive compound content and antioxidant properties of powders depend on how the source raw materials are dried [2,7,8,9]. The most commonly used method for obtaining fruit powders at present, is to dry the fruit to a low water activity level by hot air using convection drying (CD), vacuum drying, or a sublimation method, followed by grinding the dried fruit residue to the desired degree of granulation. The cheapest and most commonly used method is CD, however, this causes the most significant degradation of bioactive compounds because prolonged drying is necessary to achieve both a low water activity and moisture content in order that the fruit can be ground [6,7,9,10]. Fruits contains high levels of pectin and carbohydrates, which make the resulting particles prone to clumping, and therefore difficult to grind [6]. To achieve a water activity of approx. 0.3 by CD drying so that the fruit can be micronized into powders, usually depends on the drying conditions and temperatures used; normally taking 11 to 23 h [10]. A too intensive removal of water leads to hard shells forming on the fruit surface, thus making removal of capillary water difficult from the deeper layers within the fruit (own observations). Drying at lower temperatures, i.e., at 50 °C instead of 70 °C, significantly extends the drying time. This usually leads to a greater degradation of bioactive ingredients in fruit berries, mainly seen as greater losses of polyphenols, because the lowered drying temperature is much closer to the optimal temperature at which the degrading enzyme polyphenol oxidase functions (i.e., 40 °C) [11].

The sublimation method of drying however ensures that higher levels of bioactive compounds are preserved intact along with the fruit’s antioxidant potential in powders derived thereof. Nevertheless, this method is several times more expensive than other approaches [12]. There are many published studies investigating the feasibility of using various combinations of various drying methods, such as microwave hot air drying, microwave vacuum drying, and pulsed fluid bed dryer [13,14,15], but in the main, serious economic considerations preclude their wider use.

The present authors have recently studied whether a strong and hot airflow FBJD (fluidized-bed jet milling and drying) method previously used for the grinding of hard materials, can induce both drying and grinding of plant raw materials to form powders with the desired granulation [2]. Both grinding and drying are carried out in this method at below <50 °C for a few short minutes, thereby allowing bioactive compounds to be highly preserved. This also results in reduced particle size owing to particle-to-particle collisions in a gas stream localized at the gas nozzle, thereby enabling maximum amounts of fruit to be dried and simultaneously ground. This method is currently being tested and could soon become widely used in industrial production. Our studies so far show that this method allows powders to be obtained from hard texture materials. Raw materials of high water and pectin content require prior drying, which prevents sticking during grinding to achieve a powder consistency.

This study is focused on finding out whether powders can be obtained from fruit with sticky textures that are difficult to dry, through combining the FBJD method with pre-drying the fruit by CD, in order that the bioactive and antioxidant properties are highly retained as well as ensuring adequate microbiological and sensory qualities. The CD pre-drying to a sufficient water activity, reduced sticking between particles, allowing the ensuing FBJD method of simultaneous drying and grinding to obtain powders of the desired granulation. These procedures were performed on chokeberries; a representative fruit of those ‘fruit berries’ with textures difficult to dry, but which contain high amounts of bioactive compounds with antioxidant activity. 

## 2. Materials and Methods

### 2.1. Materials

**Figure 1:**
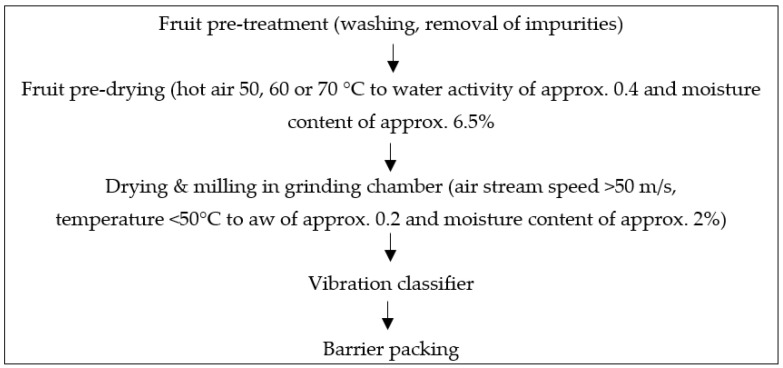
Schematic representation for preparing powders using the FBJD (fluidized-bed jet milling and drying) method.

Test samples were powders obtained from chokeberries (of the *Aronia melanocarpa* plant) gathered during the plant’s collective maturity period at the end of August 2019, which were directly delivered by a local producer. The fruit was delivered to the production plant immediately after harvesting in three batches (15 kg each), corresponding to each of the test drying temperatures, at one-day intervals; depending on the operating time of the dryer. Transport time was about one hour at a temperature of approx. 20 °C. The fruit was divided into two, one for drying using the FBJD in conjunction with the pre-drying CD method, whilst the other for just the CD method alone. The fruit was subjected to a pre-treatment process after about 3 h from being delivered to the plant, consisting of washing with water without any disinfectants and a controlled removal of residual impurities. After draining off the water, the fruit was transferred to a convection drying room and subjected to preliminary drying. Three pre-drying temperatures were selected of 70, 60, and 50 °C to test the efficiency of the process and to achieve a water activity (a_w_) and moisture content of around 0.4 and 6.5%, respectively. The CD pre-drying was carried out in an industrial dryer of the Admor company’s own design with a circular airflow and mechanical ventilation for sucking air at a speed of >3 m/s, thereby permitting the afore-mentioned a_w_ and moisture contents to be attained in a shortened time of respectively 8, 10, 12 h at 70, 60, and 50 °C. The fruits were next subjected to a final drying and a simultaneous milling using the FBJD method (Figure 1) performed at the experimental industrial manufacturing plant of the Admor Company (Radom, Poland). The device consisted of a grinding chamber installed perpendicularly in the lower part of the mixers with blades rotating at high speed allowing floating of the material when introduced into the device. This process was facilitated by an installed compressor that automatically controlled air flow rates and temperature with the latter being adjusted to the set micronization temperature while water was being removed from the particulate material. A low-temperature drying was used in these studies at a temperature of around 40 °C of the dried micronized material as measured in the fluidized bed at an air flow rate of over 50 m/s. The raw material particles were crushed and dried through mutual particle collisions in the high energy stream of air. The dried powders, with a water content of about 2%, were passed by the air stream towards a vibration classifier in order to obtain powders of the predetermined fragmentation size of <315 µm. The calibrated product was then transferred to the packaging machine. The process yield was 50 kg/h. After completing the drying process, powders of specific sizes were packed into barrier packaging ready for examination. 

The CD powders were obtained by drying the fruit using CD with air circulation (speed app. 1.5 m/s) (SUP-200, Wamed Company, Warsaw, Poland) at an air temperature of 70 °C to give an a_w_ of approx. 0.2. The post-CD dried material was then ground to a powder using a grinder with grinding knives, (MKM 6003, Bosch, Stuttgart, Germany), to give a granulation size below 315 μm.

The FBJD and CD powders, (at sizes of 315–100 μm), were selected for analysis. Classification of powder particles was performed by a vibratory sieve shaker (AS 200, RETSCH GmbH & Co., Haan, Germany) equipped with the following sieve mesh sizes: 630, 315, and 100 μm.

### 2.2. Methods

#### 2.2.1. Water Activity (a_w_)

Measurement of water activity was determined by the ratio of water vapor pressure above the solution’s surface to that of chemically pure water. A manual AquaLab Water Activity Meter was used to perform these measurements in triplicate (version 5; Decagon Devices, Inc., Pullman, WA, USA).

#### 2.2.2. Dry Matter Content/Moisture Content

The dry matter/moisture content was determined gravimetrically in the tested powders according to the AOAC method (2002) [16]. The weighing vessels were weighed, filled with powder samples, and reweighed, then placed into a drying oven (SUP 200W, Wamed, Warsaw, Poland) until a constant weight had been achieved. The samples were weighed on an analytical balance to a precision of 0.001 g. The material’s moisture content was calculated from the difference in masses. Measurements were performed in triplicate.

#### 2.2.3. Particle Classification

Particle classification of powders was performed by a Vibratory Sieve Shaker (AS 200, RETSCH GmbH & Co., Haan, Germany) equipped with sieves of 315 and 100 μm meshes.

#### 2.2.4. Preparation of Extracts for Analysis of Antioxidant Properties and Total Polyphenol Content

First, 250 mg of the each powder sample was weighed to a precision of 0.001 g (AS 220/X, Radwag, Radom, Poland) into plastic falcone tubes with a screw top (50 mL capacity) to which 25 mL of a 30:70 water-methanol mixture was added, followed by vortex shaking for 60 s (Wizard Advanced IR Vortex Mixer, VELP Scientifica Srl, Usmate, Italy). Samples were next incubated in a shaking incubator in 30 °C (IKA KS 4000i Control, IKA Ltd., Warsaw, Poland) for 60 min followed by renewed vortex shaking for 60 s to ensure thorough mixing. The mixture was then centrifuged for 15 min at 4 °C and 10,000 rpm (MPW-380 R, MPW Med. Instruments, Warsaw, Poland). The supernatant was collected for determining the antioxidant capacity and total polyphenol content.

#### 2.2.5. Determination of Antioxidant Properties

Antioxidant activity of chokeberry extracts was assayed by the ABTS+• (2,2′-azino-bis (3-ethylbenzothiazoline-6-sulphonic) acid) (Sigma-Aldrich, Poznań, Poland) radical cation assay according to the modified Re et al. (1999) method [17]. A fixed quantity of the tested extracts’ solution, (according to a pre-established dilution scheme), was aliquotted into 10 mL glass test tubes after which 3.0 mL was added of radical cations ABTS+• in PBS (Phosphate Buffer Solution, Sigma-Aldrich, Poznań, Poland). Absorbances were measured exactly after 6 min of sample incubation at about 20 °C and 734 nm wavelength, on a spectrophotometer (UV/Vis UV-6100A, Metash Instruments Co., Ltd., Shanghai, China). Results were given in µM TEAC (Trolox Equivalent Antioxidant Capacity)—the amount of Trolox µM per 1 g of the tested material’s dry mass (d.m.) content. Six replicates of each measurement were performed.

#### 2.2.6. Total Polyphenol Content

Total polyphenol content was measured by the reaction between polyphenol compounds with Folin–Ciocalteu and sodium carbonate reagents (Sigma-Aldrich, Poznań, Poland) using the modified method of Singleton and Rossi [18]. A fixed volume of powder extract solution was aliquotted into 50 mL graduated flasks, (according to a dilution scheme), followed by adding 2.5 mL of Folin–Ciocalteu reagent and 5.0 mL of 20% sodium carbonate. Distilled water was finally added up to the mark. Samples were next incubated for 60 min at about 20 °C and protected from light. Absorbances were measured at a 720 nm wavelength using a spectrophotometer (UV/Vis UV-6100A, Metash Instruments Co., Ltd., Shanghai, China). Results were expressed as mg GAE (Gallic Acid Equivalent), i.e., mg of gallic acid per 1 g of the powder’s dry mass (d.m.). Six replicates of each measurement were performed.

#### 2.2.7. Content and Separation of Individual Polyphenolic Compounds

The composition of individual polyphenols was determined using an HPLC (high-performance liquid chromatography) method described by Hallmann (2017) [19]. A weighed amount of powder (100 mg) was placed into a plastic test tube, followed by adding 1 mL of 1% ascorbic acid in methanol. This solution was thoroughly vortexed and incubated in an ultrasonic bath (15 min at 30 °C) and then spun at 5000 rpm. A 1 mL of aliquot of supernatant was collected and respun at 12,000 rpm from which a 500 µL aliquot of supernatant was removed for HPLC separation that allowed polyphenols to be thus quantitated. A reverse phase HPLC was performed on a Synergy Fusion-RP 80i column (250 × 4.60 mm), with an isocratic flow of two eluant mobile phases, acetonitrile/deionized water (55% and 10%) at pH 3.00. Run time was 36 min at a 1 mL min^−1^ flow rate and a 250–370 nm wavelength detection range. External standards (Sigma-Aldrich, Poznań, Poland) were used to identify the polyphenols. The HPLC instrument was a Shimadzu HPLC (USA Manufacturing Inc., Waltham, MA, USA), consisting of two LC-20ad pumps, a CMB-20a system controller, SIL-20acautosampler, UV/VIS SPD-20av detector, and a CTD-20ac controller. Results were expressed as mg of an individual polyphenol per 1 g of powder dry mass (d.m.). Runs were performed in triplicate.

#### 2.2.8. Vitamin C

Vitamin C was measured as the sum of ascorbic and dehydroascorbic acids using Waters HPLC (Waters, Milford, MA, USA) with UV detection at a 245 nm wavelength and mobile phase flow rate of 0.8 mL/min. A sample extraction was first performed for total vitamin C in the presence of dithiothreitol for maintaining the reduced form. The separation system consisted of a UV 2487 detector, and a RP Symmetry C18.5 µm, 4.6 × 150 mm column at a temperature of 25 °C, where the injection volume varied between 10–30 µL. Results were expressed as mg of vitamin C per 1 g of dry mass (d.m.). Runs were performed in triplicate.

#### 2.2.9. Microbiology

Microbiological analysis was performed as described in PN-EN ISO 4833-1:2013-12 [20] where the total number of colonies were counted of: temp. 30 °C, 3 days, plunge inoculation, substrate—nutrient agar whilst procedures described in PN-ISO 21527-2:2009 [21] were used to count the numbers of yeast and mould colonies at conditions of: temp. 25 °C, 5 days, plunge inoculation, selective medium—agar with chloramphenicol).

#### 2.2.10. Sensory Analysis

Sensory assessments of the CD powder samples (two replicates) were conducted by an eight-person panel of qualified experts consisting of women aged 35–55 with many years of experience in conducting sensory assessments. Panel members were highly experienced in such sensory methods; both in theoretical and practical terms.

The sensory characteristics of the CD dried fruit powders to different a_w_ and moisture content were determined by the scaling method according to ISO PN-EN ISO 13299:2016-05 (2016) [22]. Four quality parameters were selected to evaluate the powders: susceptibility to pulverising, flowability, clumping, and homogeneity of particles.

The sensory characteristic of drinks prepared from the FBJD and CD powder samples was determined by the scaling method according to ISO PN-EN ISO 13299:2016-05 (2016) [22]. Drinks were prepared by mixing 50 g of each powder with 50 g sugar per kg water followed by pasteurization at 80 °C for 30 min. Five quality parameters were selected for evaluation: fruity flavor and odor, color, smoothness, and overall quality.

The intensity of the aforementioned parameters was assessed by an unstructured 10-point scale in contractual units (cu.). Ratings were performed at the Laboratory of Sensor Analysis which meets all the criteria specified in the BS EN ISO 8589:2010 (2010) standard [23].

#### 2.2.11. Instrumental Color Measurement

Instrumental color measurement of FBJD and CD powders samples was performed by the computer image analysis using the “electronic eye” color analyzer (visual analyzer 400 IRIS, Alpha M.O.S., Toulouse, France) combined with data processing software (Alpha M.O.S., Toulouse, France). Photos of the samples were taken in a closed chamber (420 × 560 mm), which guarantees controlled light conditions, without the influence of external light on the visual analysis. Both top and bottom lighting, preventing the shadow effect (2 × 2 LED assembly lights, 6700 °K color temperature and IRC = 98, near D65: daylight during a cloudy day at 12 AM) was applied. The Basler (acA2500-14gc; Basler AG, Ahrensburg, Germany) camera with lens of a 16 mm diameter was used to take the pictures of the samples. After calibrating the instrument with a certified color scale, the samples were placed in a removable white tray, diffusing uniform light inside the device’s lockable light chamber. Measurements were made in the CIE L*a*b* system (*L*-brightness, *+a*-red, *-a*-green, *+b*-yellow, *-b*-blue) and RGB system (R–red, G–green, B–blue). The values for parameters L*, a*, b*, R, G, B were calculated as weighted averages, taking into account the frequency of appearance of individual color code. Measurements were performed in triplicate.

#### 2.2.12. Statistical Analysis

Statistica 10.0 (Statsoft) software was used for all statistical processing. A one-way-analysis of variation (ANOVA) was used to test for significant differences in the quality characteristics between the compared powders, using the Duncan significance test for post-hoc testing between groups at *p* < 0.05. Obtained results are presented in tables and figures as average values (for the color parameter results, a weighted average was calculated) (×) with standard deviations (SD). Statistical outcomes are also shown in figures and tables using appropriate letters of the alphabet to define homogeneous groups.

## 3. Results and Discussion

The first stage of the study concerned the microbiological quality and the content of bioactive compounds found in chokeberry powders when prepared by the FBJD method after pre-drying the fruit with the CD method at temperatures of 50, 60, and 70 °C to obtain a water activity of approx. 0.4 and moisture content of about 6.5% (Table 1). Samples tested for microbiological content were pre-dried fruits as well as powders obtained after applying the FBJD method, i.e., analytes measured were polyphenols and vitamin C contents along with antioxidant properties.

The second stage of the study focused on comparing the FBJD powders obtained after pre-drying at 70 °C with those obtained by the CD method alone. The latter consisting of drying fruits at 70 °C to a consistency that allows them to be crushed (a_w_ of approx. 0.2) and then micronized to a similar particle size as the FBJD powders. The qualitative parameters assessed were polyphenols and vitamin C content along with antioxidant properties, with due regard to values characteristic for fresh fruit as well as color and sensory properties. Details of the tested powders are presented in Table 1.

### 3.1. Comparison of the Microbiological Quality and Bioactive Compounds’ Content in Chokeberry Powders, Obtained by the FBJD Method, with Fruit Pre-Drying at Different Temperatures

Whenever powders are prepared from fruit berries with high contents of water and pectin, then, the direct FJBD is not recommended because of particle clumping during drying, as in the case of hard-textured materials. Such samples should thus be pre-dried in order to prevent sticking during the later stages of simultaneous milling and drying. A significantly reduced drying time of 8 and 12 h, (at respectively 70 and 50 °C), was found when using the FBJD method with those chokeberries pre-dried to an a_w_ of approximately 0.4, followed by further drying with simultaneous grinding, (lasting only about 10 min), compared with the CD method alone of 28 h in 70 °C. This had a large effect on the quality of the resulting powders. In this study, chokeberries were dried by hot air using the CD method at temperatures of 50, 60, and 70 °C, so that a water activity of about 0.42 was achieved, the pre-dried fruits then being further dried and milled with the FBJD method to achieve a water activity of approx. 0.2.

#### 3.1.1. Microbiology Quality of FBJD Powders Obtained from Fruits Subjected to Pre-Drying at Different Temperatures

There was a large diversity observed in the amounts of microorganisms in both pre-dried fruits and the resulting powders (Table 2), expressed as both the total number of colonies (at 30 °C) and the forms of yeast and moulds. Lower amounts were found in those chokeberry fruit that were pre-dried which may reflect the more favorable conditions for microbial propagation during simultaneous drying and milling (at 50 °C with a strong air stream) [24]. Pre–drying of the chokeberry fruits to water activity levels of 0.40–0.42 reduced the yeast and mold content to <10 CFU g^−1^ at 60 and 70 °C and also significantly reduced the overall microbial content. Chokeberry fruits dried at 50 °C had higher yeast and mold contents, which can be explained by a slower transfer of heat to the inside of the dried fruits and that an optimal temperature for microbial growth was maintained for a longer time. The total number of colonies was reduced (from 3 to 4-log redaction) in the dried chokeberry fruits and powders obtained using both the FJBD and CD method, when compared to raw fruit where the yeast and mould content was <10 CFU g^−1^. These values were significantly lower with the FBJD method with pre-drying; a 2–3-log reduction for general content and 1–3-log reduction for yeast and mold.

A study by Witthuhn et al. (2004) [25], on dried fruits, found acceptable levels of microorganisms similar to ones in this study. Fruit should be pre-treated before drying by disinfection methods used in industry to achieve lower microbial contents during powder production. A method that is now increasingly applied is using ozone; both in gaseous and aqueous forms [26]. During storage, powders should be barrier packed to maintain a sufficiently low water activity, which is very important to ensure the powders’ microbiological stability. A water activity of less than 0.6 is considered acceptable for raw products since microbiological activity, including osmophilic yeast, is reduced [10]. Water activity had to be much lower (approx. 0.2), in the tested powders, in order to provide a suitable and non-sticky texture, which further contributed to the microbial stability of the powder so obtained.

#### 3.1.2. The Content of Bioactive Compounds in Raw Chokeberry and in Chokeberry FBJD Powders Obtained from Fruits Subjected to Pre-Drying at Different Temperatures

Table 3 shows the vitamin C and total polyphenols contents together with the antioxidant activity in raw chokeberry and in chokeberry FBJD powders prepared with CD pre-drying at different temperatures (50, 60, and 70 °C). The content of vitamin C was determined using the HPLC method, whilst the content of polyphenols and antioxidant properties were spectrophotometrically determined. Much lower levels of bioactive ingredients were found at all drying temperatures when comparing the obtained powders with raw chokeberry fruit. Significantly lower amounts of vitamin C, polyphenols, and antioxidant activities were found in powders which were prepared by the CD pre-drying at 50 °C than at 60 and 70 °C. Similar results had been obtained in a study by Samoticha et al. (2016) [10] where chokeberries were dried at 50, 60, and 70 °C by convection with water activities ranging from 0.330 to 0.336. This study attributed the greater losses at 50 °C in polyphenols and lower antioxidant properties to the proximity of optimal temperature at which the degrading enzyme polyphenol oxidase functions (40 °C). According to Lopez-Nicolas and Garcıa-Carmona (2010) [11], losses in polyphenols during CD are linked to enzymatic and non-enzymatic oxidation. Rapid heating of short duration may inactivate oxidative enzymes and thus phenolic compounds are better preserved. A study by Arancibia-Avila et al. (2012) [27] showed no losses of polyphenols in blueberry fruit samples obtained during heating at 100 °C for 20 min. Nevertheless, both our study and one by Samotich et al. (2016) [10], carried out fruit heating at lower temperatures. Changes in polyphenolic content were greater during drying at 50 °C, than drying at 70 °C, where the drying time was about 50% shorter in the latter than drying at 50 °C. Another reason why polyphenols and vitamin C degrade rapidly at the lower drying temperatures may be due to the longer drying times at 50 °C as compared to 70 °C [10,28]. Higher antioxidant activity in FBJD fruit generated powders dried at higher temperature can not only be due to the shorter pre-drying time of fruits, but because Maillard reaction products may be formed (and caramelization) at higher temperatures, which also exhibit antioxidant properties [29].

The sum total of individual polyphenols determined by the HPLC method (Table 4) was lower than the amount of polyphenols determined spectrophotometrically. This indicates that not all polyphenols were determined by HPLC, whilst other phenolic containing compounds had been picked up by spectrophotometry, thus overestimating the result [30]. The polyphenol content was highest (*p* < 0.05) in powders obtained from fruits dried at 70 °C, and significant differences (*p* < 0.05) were also found for powders after pre-drying at 60 and 50 °C. Particularly, large differences were observed in phenolic and flavonol content between the tested temperatures whereas differences were insignificant for total anthocyanin contents (*p* < 0.05). The highest levels of cyanidin-3,5-di-O-arabinoside were however noted at the drying temperature of 70 °C, but lowest at 50 °C. There is no data in the literature on the contents of specific polyphenols in powders according to drying temperatures of fruits. Various statistically significant (*p* < 0.05) differences in polyphenols were observed in all studied groups when comparing the FBJD—prior drying procedure (at the three temperatures), with raw fruit; the greatest differences being between powders obtained by the FBJD method with fruit drying at 50 °C (Table 4). There were also statistically significant differences (*p* < 0.05) found between the total content of individual polyphenols (i.e., phenolic acids, flavonoids, flavonols), except anthocyanins, in the studied powders obtained at different drying temperatures.

### 3.2. Comparison of Powders Obtained by the FBJD Method with Pre-Drying of Fruits at 70 °C with those Obtained by Drying Chokeberry Fruits Using CD

The research included determining the a_w_ at which it is possible to micronize the chokeberry fruit and comparing the content of bioactive components, antioxidant properties, sensory quality, and color of the pre-drying FJBD powders with the powders obtained by the CD method.

#### 3.2.1. Comparing the Sensory Quality of CD Powders Prepared by Drying Fruits to Different a_w_ and Moisture Content

**Figure 2:**
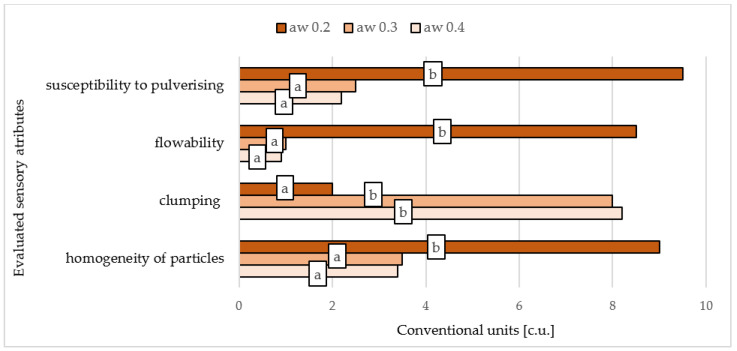
Sensory characteristics of the dried chokeberry using convection drying to different water activity (a_w_), subjected then to milling process. Values are means ± standard deviation. a,b—different letters in the same line are significantly different (Duncan’s test, *p* < 0.05).

**Figure 3:**
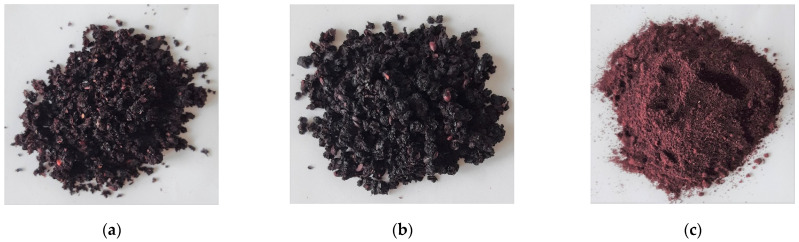
Chokeberry fruits crushed to a powder form, dried using the CD (convection drying) method to a_w_: (**a**) 0.4, (**b**) 0.3, and (**c**) 0.2.

Figure 2 shows the results of the sensory evaluation of chokeberry powders obtained by drying fruits with hot air (CD) to various levels of water activity and then subjected to comminute. Figure 3 shows chokeberry fruits crushed to a powder form, dried to three different a_w_. We thus conclude that the drying process must be conducted until water activity becomes <0.2 in order to obtain powders of an adequate texture (flowability) from fruit berries, which contain sugars that affect hygroscopicity and viscosity [31]. Milled fruits have a sticky structure at higher levels of water activity, which impedes obtaining an optimal and pulverized texture. To achieve water activity levels below 0.2 requires a much longer time than when pre-drying is used where free water is mainly removed. Therefore, in the case of convective drying of fruit intended for grinding into powder form, up to several hours of drying of fruit are required to achieve a_w_ < 0.2.

#### 3.2.2. Comparing the Content of Bioactive Compounds, Sensory Quality, and Color of Pre-Dried Powders (at 70 °C) Obtained by the FBJD Method with the Powders Obtained by the CD Method at 70 °C

**Figure 4:**
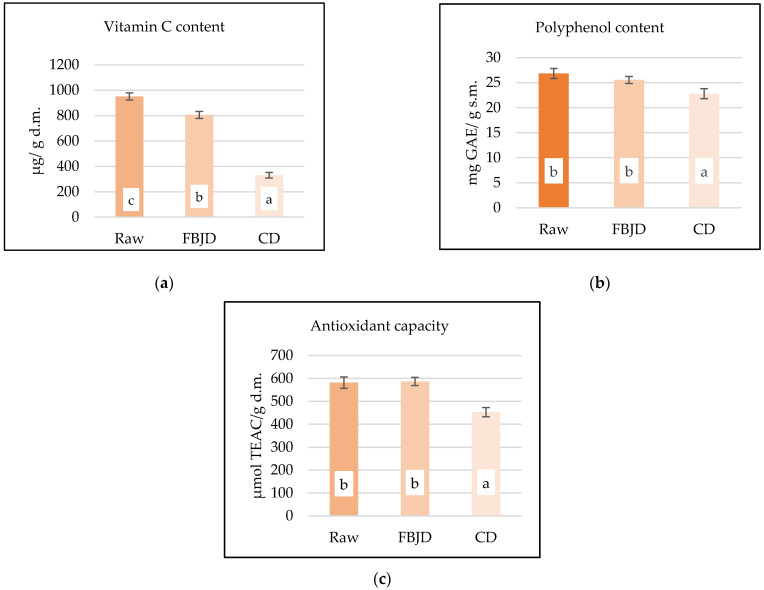
Vitamin C (**a**) and polyphenol (**b**) contents and antioxidant properties (**c**) of chokeberry and chokeberry powders obtained by the CD method in 70 °C to a_w_ of approx. 0.2 and FBJD method using pre-drying at 70 °C. Values are means ± standard deviation (*n* = 3); a,b—different letters in the same line are significantly different (Duncan’s test, *p* < 0.05),FBJD—fluidized-bed jet milling and drying; CD—convective drying; GAE—gallic acid equivalent; TEAC—Trolox equivalent antioxidant capacity; d.m.—dry mass.

The powders obtained by the FBJD method had a significantly higher content of bioactive compounds (polyphenols, vitamin C) and antioxidant properties than the CD-derived powders (Figure 4a–c). Significant losses (*p* < 0.05) of vitamin C were also observed between raw chokeberry and the powders, with losses of 15 and 65% for FBJD and CD powders, respectively (Figure 3a). A previous study by the authors demonstrated that vitamin C contents were significantly reduced by approximately 29% in chokeberry powders obtained through the FBJD method compared to fresh fruit samples [2]. When compared to the present study, such different outcomes in the FBJD-obtained powders may have arisen because of the lower pre-drying temperature of 50 °C in the 2017 study as compared to 70 °C used in the present study. Higher amounts of total polyphenolic content and antioxidant properties were preserved in the FBJD powders than in CD powders (Figure 4b,c). No significant (*p* < 0.05) differences were found in polyphenol content and antioxidant activity between FBJD powders and raw fruit. A previous study by our group [7,8] showed similar differences in polyphenol content and antioxidant activity between FBJD and CD powders (14% and 17%, respectively in Sadowska et al. (2019) [7] vs. 11% and 23% in the current study).

#### 3.2.3. Comparing Sensory Quality and Color of CD and FBJD Powders

**Figure 5:**
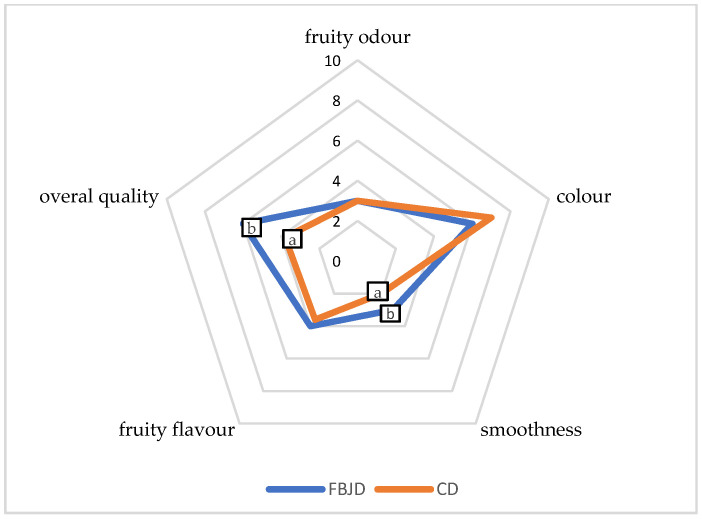
Comparison of the sensory quality of the drinks obtained by using the FBJD powders (method combined with pre-drying of fruits at 70 °C to a_w_ 0.4) with the drinks obtained by using the CD powders (70 °C, a_w_ 0.2). Values are means ± standard deviation; a,b—different letters in the same line are significantly different (Duncan’s test, *p* < 0.05); FBJD—fluidized-bed jet milling and drying; CD—convective drying.

The FBJD powder drinks prepared had significantly (*p* < 0.05) higher smoothness and overall quality than did the CD ones (Figure 5). Such differences may have been due to how the fruit was ground into its powder form, i.e., the breaking up of partially dried fruits in a fluidized-bed under a strong air stream compared with the grinding of the fruits dried with the CD method to a water activity of 0.2 using a device fitted with cutting knives.

**Figure 6:**
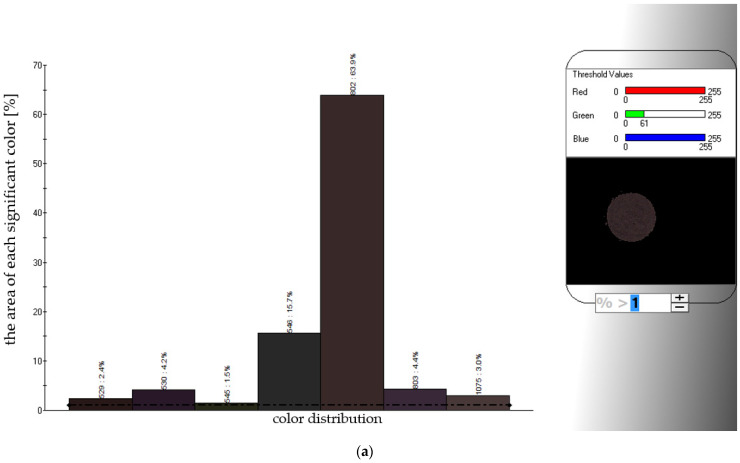

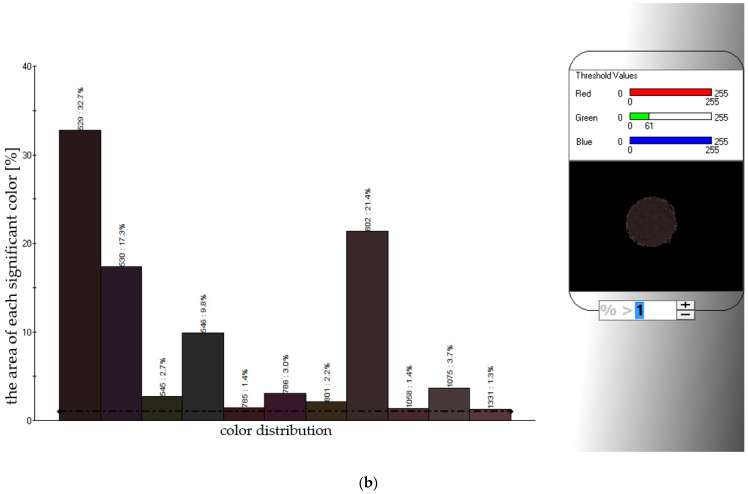
Color analysis of the FBJD (**a**) and CD (**b**) powders using the instrumental method (‘electronic eye’). FBJD—fluidized-bed jet milling and drying; CD—convective drying. The dashed line marks the area (>1%) from which each significant color was taken into account.

The analysis of color, with the ‘electronic eye’ instrument, is based on taking pictures of the samples. The electronic eye is a computer vision technology that transforms images into digital images. The e-eye uses image sensors instead of human eyes to collect images of objects, and uses a computer simulation criterion to identify the image to prevent subjective deviation of human eyes. The evaluation of measurements is performed with the use of software that creates color spectra with the indication of the size (expressed as a percentage) of their presence in the tested sample [32]. The images are then processed as a color spectrum, with the area of each significant color marked as a percentage (Figure 6a,b). Various shades of brown to dark brown were observed, in different proportions, in the tested samples of chokeberry powders obtained by the FBJD and CD method; depending on the drying method used. Significant differences in almost all color parameters (except the b* parameter) were noted between FBJD and CD powders (Table 5) in significantly (*p* < 0.05) brighter colors, (L*a*b* color space) with a significantly (*p* < 0.05) less intense shade of red but of similar yellow hue compared to the CD powders. The FBJD powders had significantly (*p* < 0.05) more intense tones of all three colors than CD powders when taking into account the RGB color model. The color of the droughts is an important parameter when choosing a drying method, because it may provide information on physicochemical changes that had occurred during the drying and also during the grinding processes. The color change is related to, among others, a decrease in the content of anthocyanins [33]. We thus conclude that a longer convective drying time produced powders with a darker shade (CD powders), whilst those from the FBJD method possessed a more intense individual color shade. The FBJD powders also showed a higher level of polyphenol preservation (including anthocyanins) (Figure 4b), which may correspond to their more intense color. The differences in the color of the powders may also be related to the production of dark substances during convection drying, i.e., the products of the Maillard reaction. In a study by Coimbra et al. (2011) [34], obtained results allow affirming that the drying process (sun-drying direct exposure to solar radiation and a hot air drying (40 °C, 7 days)) of pears promotes the occurrence of Maillard reaction, contributing to the characteristic reddish brown color of the final product. While Piga et al. (2003) [35] studies showed presence of hydroxymethylfurfural (advanced Maillard reaction product) in dried plums in the temperature range of 60–85 °C. This greater intensity of color in FBJD powders may indeed be very beneficial whenever applying these type of powders as coloring additives. Previous studies by the authors showed that FBJD powders were redder and brighter when compared to freeze-dried ones [2].

This study has thereby demonstrated that a new, instrumental method of color measurement, (using an ‘electronic eye’ with L*a*b* and RGB color spaces), allows determination to be made for more accurate and significantly broader color characteristics of products than using a reflectance method with only L*a*b* color spaces. This is because such measurements can be used to obtain differences in terms of colors and color distribution, as well as a high variability in the visual aspect of some products [36].

## 4. Conclusions

This study has plugged some of the knowledge gaps in terms of those widely used methods employing high energy air streams (jet systems); not only for micronizing hard-structured materials, (e.g., minerals, pharmaceutical products), but also food products with a hard texture without the need for any prior drying, as well as plant materials with a sticky structure, where initial pre-drying is advisable.

The FBJD tested method for preparing fruit powders, involves simultaneous drying and milling at temperatures of <50 °C of samples pretreated with hot air drying. This allows high quality fruit powders to be obtained and also significantly shortens their final drying times when compared to the CD method. The FBJD method is especially useful for drying and grinding hard textured material. Fruits having a high moisture content and sticky texture, such as the chokeberry and other fruit berries, should be pre-dried until the appropriate texture is obtained. More advantageous bioactive properties were found, (i.e., higher antioxidant properties, higher vitamin C, and total polyphenol content), when fruit was pre-dried at the higher temperatures of 60 and 70 °C compared to 50 °C; which may have arisen from a more rapid inactivation of oxidative enzymes along with shorter drying times at the higher temperatures. Pre-dried FBJD powders (at 70 °C) had higher contents of marked bioactive compounds (polyphenols and vitamin C content) and more favorable sensory properties compared to the CD powders. The FBJD method combined with CD pre-drying compares more favorably to the CD method in obtaining dried fruits with a higher vitamin C preservation of 84% (CD powders 35%), higher total polyphenol content of about 12%, together with higher antioxidant activity (of about 10%). We have demonstrated that the FBJD method, combined with CD pre-drying, allows high quality powders to be prepared in a much shorter time in those fruits with difficult to dry and mill textures compared to just using CD alone. This may therefore significantly reduce production costs and may prove to be a very competitive alternative to other commonly used methods of obtaining fruit powders. Such decisions need to be taken based on accurate assessments before launching into any larger scale production. A limitation of this study is that the FBJD method is not widely available at the moment, where both micronization and drying of the material in a fluidized bed is required. Nevertheless, this method is of great interest to food producers and it should be expected to be widely disseminated in the near future whilst at the same time, further scientific research can be conducted.

## Figures and Tables

**Table 1:** Tested samples characterization.

Pre-Drying Temperatures			50 °C	60 °C	70 °C
Fruits pre-dried using CD method	water activity		0.4 ± 0.0	0.4 ± 0.0	0.4 ± 0.0
	moisture content (%)		6.5 ± 0.1	6.4 ± 0.1	6.5 ± 0.0
	pre-drying time (h)		12	10	8
Powders obtained by the FBJD method after pre-drying the fruits using CD method	water activity		0.1 ± 0.0	0.1 ± 0.0	0.1 ± 0.0
	moisture content (%)		2.2 ± 0.0	2.2 ±0.0	2.2 ± 0.1
	drying and milling time FJBD (min)		10	10	10
	the mesh size of the screens (%)	630–315 µm	0	0	0
		315–100 µm	86.7	87.4	79.5
		<100 µm	14.1	13.9	21.2
Drying temperature			70 °C		
Powders obtained by the CD method	water activity		0.2 ± 0.0	0.3 ± 0.0	0.4 ± 0.0
	moisture content (%)		2.9 ± 0.1	3.8 ± 0.0	6.3 ± 0.0
	drying time (h)		28	20	16
	the mesh size of the screens (%)	630–315 µm	11.6	22.1	30.5
		315–100 µm	65.0	58.4	55.2
		<100 µm	24.3	20.8	15.1

FBJD—fluidized-bed jet milling and drying; CD—convective drying.

**Table 2:** Total number of colonies and amounts of yeast and mould in whole chokeberry fruits and after initial pre-drying at different temperatures (50, 60, and 70 °C) and in powders obtained by the FBJD method after pre-drying fruit at and in those the same temperatures.

Samples	Microorganisms (CFU g^−1^)	Dried Samples Pre-Drying Temp.			Raw Sample
		50 °C	60 °C	70 °C	
Fruits raw and pre-dried to water activity of 0.4 using CD method	total number of colonies	1.0 × 10^3^	1.4 × 10^3^	4.8 × 10^2^	1.1 × 10^6^
	yeast	4.0 × 10^2^	<10	<10	3.0 × 10^4^
	mould	5.0 × 10	<10	<10	2.0 × 10^3^
Powders obtained by the FBJD method after pre-drying the fruit to water activity of 0.4	total number of colonies	1.9 × 10^4^	2.6 × 10^4^	2.4 × 10^3^	
	yeast	2.2 × 10^3^	1.0 × 10^2^	50	
	mould	7.5 × 10^2^	80	41	

FBJD—fluidized-bed jet milling and drying; CD—convective drying; CFU—colony forming unit.

**Table 3:** Vitamin C, determined using HPLC method, and polyphenolic contents along with antioxidant properties, both determined by spectrophotometric method, in chokeberry powders prepared by the FBJD method with pre-drying fruit at temperatures of 50, 60, and 70 °C.

Bioactive Compounds/ Antioxidant Properties		Dried Samples Pre-Drying Temperatures			Raw Sample
		50 °C	60 °C	70 °C	
Vitamin C content	µg/g d.m.	298.1 ± 40.6 ^a^	505.2 ± 71.7 ^b^	805.6 ± 80.3 ^c^	950.6 ± 95.7 ^d^
Polyphenols content	mg GAE/g d.m.	19.6 ± 1.1 ^a^	23.0 ± 1.2 ^b^	25.6 ± 0.7 ^b^	26.8 ± 0.8 ^b^
Antioxidant properties	µM TEAC/g d.m.	368.6 ± 13.2 ^a^	595.5 ± 26.2 ^b^	589.1 ± 13.2 ^b^	590.4 ± 33.1 ^b^

Values are means ± standard deviation. a–d—different letters in the same line are significantly different (Duncan’s test, *p* < 0.05). FBJD—fluidized-bed jet milling and drying; CD—convective drying; GAE—gallic acid equivalent; TEAC—Trolox equivalent antioxidant capacity; d.m.—dry mass.

**Table 4:** Individual polyphenol content (mg/g d.m.) determined using HPLC method in chokeberry FBJD powders obtained by using pre-drying at three temperatures of 50, 60, and 70 °C.

Polyphenols	Dried Samples/Pre-Drying Temperatures			Raw Sample
	50 °C	60 °C	70 °C	
Total polyphenols	19.1 ± 0.2 ^a^	20.2 ± 0.2 ^b^	20.8 ± 0.1 ^c^	23.3 ± 0.2 ^d^
Total phenolic acids	1.6 ± 0.1 ^a^	2.2 ± 0.2 ^b^	3.0 ± 0.1 ^c^	4.6 ± 0.1 ^d^
gallic acid	0.1 ± 0.0 ^b^	0.0 ± 0.0 ^a^	0.0 ± 0.0 ^a^	1.6 ± 0.0 ^c^
chlorogenic acid	1.2 ± 0.9 ^a^	1.4 ± 0.9 ^a^	1.3 ± 0.1 ^a^	1.6 ± 0.0 ^b^
caffeic acid	0.0 ± 0.0 ^a^	0.0 ± 0.0 ^a^	0.0 ± 0.0 ^a^	0.0 ± 0.0 ^a^
p-coumaric acid	0.0 ± 0.0 ^a^	0.8 ± 0.1 ^b^	1.7 ± 0.1 ^c^	1.3 ± 0.1 ^c^
ferulic acid	0.2 ± 0.0 ^b^	0.1 ± 0.0 ^a^	0.1 ± 0.0 ^a^	0.1 ± 0.0 ^a^
Total flavonoids	17.5 ± 0.3 ^a^	18.0 ± 0.3 ^b^	17.7 ± 0.1 ^a,b^	19.4 ± 0.2 ^c^
Total flavonols	2.0 ± 0.0 ^a^	2.4 ± 0.3 ^b^	2.3 ± 0.1 ^a,b^	4.0 ± 0.1 ^c^
epigallocatechin gallate	1.1 ± 0.1 ^a^	1.3 ± 0.3 ^a^	1.3 ± 0.2 ^a^	2.7 ± 0.1 ^b^
quercetin-3-O-rutinoside	0.5 ± 0.0 ^a^	0.6 ± 0.0 ^a^	0.5 ± 0.0 ^a^	0.5 ± 0.0 ^a^
myricetin	0.1 ± 0.0 ^b^	0.0 ± 0.0 ^a^	0.0 ± 0.0 ^a^	0.0 ± 0.0 ^a^
luteolin	0.0 ± 0.0 ^a^	0.0 ± 0.0 ^a^	0.0 ± 0.0 ^a^	0.2 ± 0.0 ^b^
kaempferol	0.0 ± 0.0 ^b^	0.0 ± 0.0 ^a^	0.0 ± 0.0 ^a^	0.2 ± 0.0 ^c^
quercetin-3-O-glucoside	0.0 ± 0.0 ^a^	0.4 ± 0.0 ^c^	0.4 ± 0.0 ^b^	0.3 ± 0.0 ^b^
kaempferol-3-O-glucoside	0.0 ± 0.0 ^c^	0.0 ± 0.0 ^a^	0.0 ± 0.0 ^b^	0.0 ± 0.0 ^b^
Total anthocyanins	15.5 ± 0.2 ^a^	15.5 ± 0.2 ^a^	15.5 ± 0.2 ^a^	16.4 ± 0.8 ^b^
cyanidin-3,5-di-O-glucoside	14.4 ± 0.2 ^a^	14.3 ± 0.2 ^a^	14.2 ± 0.2 ^a^	14.7 ± 0.1 ^a^
cyanidin-3,5-di-O-arabinoside	1.2 ± 0.1 ^a^	1.3 ± 0.0 ^b^	1.2 ± 0.0 ^a,b^	1.7 ± 0.0 ^c^

Values are means ± standard deviation. a–d: different letters in the same line are significantly different (Duncan’s test, *p* < 0.05).

**Table 5:** Color parameters in CIE L*a*b* and RGB space obtained for chokeberry FBJD and CD powders using the instrumental method (‘electronic eye’).

Color Parameters	FBJD	CD
L* (lightness)	17.4 ± 0.3 ^b^	14.17 ± 1.0 ^a^
a* (redness)	6.5 ± 0.5 ^a^	7.9 ± 0.4 ^b^
b* (yellowness)	1.7 ± 0.3 ^a^	1.4 ± 0.1 ^a^
R (redness)	52.7 ± 0.6 ^b^	46.3 ± 0.6 ^a^
G (greenness)	38.7 ± 0.6 ^b^	31.7 ± 0.6 ^a^
B (blueness)	41.0 ± 1.0 ^b^	34.0 ± 1.0 ^a^

Values are means ± standard deviation. a,b—different letters in the same line are significantly different (Duncan’s test, *p* < 0.05). FBJD—fluidized-bed jet milling and drying; CD—convective drying.

## Data Availability

Not applicable.
